# Plant extract-based nanoemulsion for controlling sweet potato pests and weeds

**DOI:** 10.1038/s41598-025-32263-9

**Published:** 2025-12-10

**Authors:** Jarongsak Pumnuan, Anusart Ruddit, Anuwat Lakyat, Thanaporn Doungnapa, Jantra Deemak, Muanfan Thongbang, Suriyasit Somnuek, Naphat Somala, Somsak Kramchote

**Affiliations:** 1 School of Agricultural Technology, King Mongkut’s Institute of Technology Ladkrabang, Bangkok, 10520 Thailand; 2 College of Food and Hospitality Innovation, Phetchaburi Rajabhat University, Phetchaburi, 76000 Thailand

**Keywords:** *Cylas formicarius*, *Fusarium* sp., *Amaranthus viridis*, *Echinochloa crus-galli*, Star anise, Long pepper, Plant sciences, Zoology

## Abstract

The sweet potato weevil (*Cylas formicarius*) is the most destructive pest of sweet potato and is often associated with diseases and weeds. The use of plant extracts as pest control agents is a promising, eco-friendly approach, but their effectiveness often declines under field conditions. Therefore, nanoemulsion technology was applied to improve the dispersion and bioefficacy of active compounds. In this study, we aimed to develop plant extract-based nanoemulsions as multifunctional biopesticides capable of controlling insect pests, fungal pathogens, and weeds. Twenty-one plant extracts were screened for their effects adult SPW via contact methods. The most potent extracts were then formulated into nanoemulsions and evaluated for insecticidal efficacy—including contact toxicity, repellency, and oviposition inhibition. In addition to insecticidal effects, antifungal and herbicidal activities were systematically assessed against *Fusarium* sp. and two common weed species in sweet potato fields, *Amaranthus tricolor* and *Echinochloa crus-galli*. The major chemical constituents of key extracts were identified using gas chromatography–mass spectrometry (GC–MS). Among all extracts, the hexane extracts of star anise and long pepper exhibited the highest contact toxicity, with LC50 values ranging from 2.051 to 2.415% at 24 h. When formulated into nanoemulsions (NHEF-1 and NHEH-2), toxicity increased 16- to 18-fold, lowering LC_50_ values to 0.114–0.150%, and at above 0.1%, oviposition was inhibited by more than 85%. Both formulations also demonstrated significant antifungal activity against *Fusarium* sp. (44.6–56.8% growth inhibition) and completely inhibited the germination of *A. tricolor* (at 0.01%) and *E. crus-galli* (at 1.0%). GC–MS analysis revealed anethole (84.49%) as the predominant compound in the star anise extract, with long pepper extract containing moderate amounts of several compounds. These findings demonstrate the potential of plant extract-based nanoemulsions as eco-friendly, multifunctional biopesticides for sustainable sweet potato management, integrating enhanced insecticidal, antifungal, and herbicidal effects.

**Supplementary Information:**

The online version contains supplementary material available at 10.1038/s41598-025-32263-9.

## Introduction

Sweet potato weevil (*Cylas formicarius* (Fabricius): SPW) is widely regarded as the most destructive pest of the sweet potato (*Ipomoea batatas* L.) crop, which causes severe damage resulting in multimillion-dollar losses worldwide^1^. A complete life cycle spans one to two months, yielding approximately five to eight generations per year^2^. Upon hatching, SPW larvae burrow into tubers producing a bitter taste and terpene-like odor, thereby reducing quality and marketability of the tubers. Moreover, these tunnels provide entry points for soilborne pathogens, leading to secondary infections by fungi and bacteria^1^. Yield losses of 60–70%, and in some cases up to 90%, have been reported in heavily infested regions^3^.

Current SPW management heavily relies on synthetic insecticides—such as organophosphates, chlorpyrifos, and imidacloprid—that act on the insect’s central nervous system^4^. Despite their efficacy, these chemicals can leave toxic residues on agricultural products, harm non-target organisms, promote insecticide resistance, and pose environmental and human health risks^5^. Integrated Pest Management (IPM) approaches have therefore been adopted for SPW control, encompassing host-plant resistance, biotechnological methods, cultural practices, biological control, and both botanical and synthetic chemical pesticides^3^. Among these methods, botanical insecticides are particularly appealing due to their reduced environmental impact and minimal health risks^6^.

Numerous studies have investigated the potential of plant-derived substances for SPW management. For instance, Jayaprakas et al. reported that a 5% cassava seed extract achieved 86.7% SPW mortality at 24 h post-treatment^7^, while Facey et al. documented insecticidal activities of *Hyptis capitata* essential oil (LD_50_ = 55–60 µg/insect at 72 h)^8^. Williams et al. demonstrated that *Cleome viscosa* hexane extracts exerted pyrethroid-like contact toxicity, causing a 60% knockdown in adult SPWs. NeemAzal 5, a commercial neem-based bioinsecticide (0.05%)^9^, significantly suppressed SPW oviposition and feeding^10^. Similarly, *Mitragyna speciosa* leaf hexane extract at 4% yielded >70% mortality within 24 h (LC_50_ values of 3.50, 2.88, and 2.27% at 24, 48, and 72 h, respectively) while also repelling >77% of adults and inhibiting >80% oviposition^11^. Furthermore, Nicotiana tabacum leaf crude extracts resulted in complete adult mortality of 72 h (LD_50_ of 0.49 and 1.67 µg/insect), and an ethyl acetate extract of *Piper betle* showed high mortality (LD_50_ of 11.78, 17.13, and 8.68 ppm at 24, 48, and 72 h, respectively)^12,13^. Jaoko et al. likewise highlighted the potential of *Melia volkensii* extracts for SPW suppression^14^.

In addition to weevil damage, sweet potato crops face losses from microbial infections, particularly *Fusarium* species, which commonly cause dry rot and wilt. *Fusarium* wilt can lead to more than 50% yield losses^15,16^. Infected plants often exhibit stunted growth, adventitious roots, cracked storage roots with brown-to-black lesions, and premature leaf abscission. Moreover, weeds such as *Amaranthus viridis* and *Echinochloa crus-galli* frequently infest sweet potato fields, competing for nutrients and water while adapting to diverse environmental conditions^17,18^. Infestation of this weed can further exacerbate yield losses^19,20^.

Recent studies indicate that various medicinal plants exhibit concurrent insecticidal, antifungal, and herbicidal properties, making them promising multipurpose agents in crop protection^21−23^. For example, *Chromolaena odorata* extracts have shown herbicidal activity against both *A. viridis* and *E. crus-galli*, suggesting they could be used in early post-emergence weed management^21^. Nanoemulsions formulated from *Cymbopogon nardus* (citronella essential oil) also inhibited seed germination of *E. crus-galli* and *Amaranthus tricolor*, serving as potential natural pre-emergence herbicides, and its mechanism was inhibition of seed imbibition and α-amylase activity^22^. Further, extracts of *Myroxylon balsamum*, *Punica granatum*, and *Salvia guaranitica* have demonstrated significant antifungal activity against *Fusarium* spp^24^.

Despite these promising findings, many Thai medicinal plants with prospective pesticidal efficacy remain underexplored. Therefore, the objectives of this study were to (i) compare the insecticidal activities—contact toxicity, repellency, and oviposition inhibition—of Thai medicinal plant extracts (extracted with hexane, acetone, and ethanol) against adult SPWs; (ii) evaluate the insecticidal, antifungal, and herbicidal potential of nanoemulsions formulated from the most effective plant extracts; and (iii) identify the major chemical constituents of the primary plant extracts used in these formulations.

## Materials and methods

### Plant extracts preparation and chemical characterization

Four dried botanical species— star anise (*Illicium verum*), long pepper (*Piper longum*), clove (*Syzygium aromaticum*) and cinnamon (*Cinnamomum verum)*—were purchased from a traditional Chinese herbal market in Bangkok, Thailand, and authenticated prior to extraction. In addition, 17 fresh plant species—myrtle grass, bitter bush, Siamese’s Maerua, snake climber, golden shower, sasswood, tuba root, jewel vine, kratom, mint weed, weeping lantana, stemona, blue gum, heart-leaved moonseed, Siamese neem tree, Siam cardamom, and cassumunar ginger—were collected from local crop fields in the Phanom Thuan District of Kanchanaburi Province, Thailand, and also authenticated by a plant taxonomist before use. All plant materials were collected at a comparable maturity stage and prepared under uniform conditions to minimize variation in phytochemical composition among replicates. All fresh plant materials were washed thoroughly and cut into small pieces and dried in an oven at 45 °C for 48 h. The dried samples were then ground into fine powder and subjected to sequential maceration in hexane, acetone, and ethanol, following a modified protocol from Pumnuan et al.^25^. Briefly, 100 g of powder was immerged in 400 mL of solvent (1:4, w/v) for 3 days at room temperature (30 ± 5 °C). The mixture was then filtered through filter paper, Whatman™ No.1 filter paper, and the filtrates were concentrated under reduced pressure using a rotary evaporator. The residual plant material from each extraction step was subsequently used for the next solvent in the same manner, resulting in hexane, acetone, and ethanol crude extracts for each plant. All crude extracts were stored in amber bottles at 4 °C and protected from light until further use. For preliminary bioassays, the crude extracts were dissolved in a solution of Tween-20 and water (2:1 ratio of extract to Tween-20) and further diluted to final concentrations of 1–5%. The chemical profiles of selected extracts were characterized using appropriate chromatographic and spectrometric methods (e.g., GC–MS or LC–MS) to identify major bioactive constituents.

Plant Extract-Based Nanoemulsion Formulas derived from plant hexane extract-based nanoemulsions (NHEs) were prepared following the methodology of Doungnapa et al.^26^. Specially, the hexane extract of star anise (designed NHE-S) was formulated using ratios of extract : surfactant (NP9):co-surfactant (Tween-20) equal to 1:3:1, while that of long pepper (NHE-L), the ratio was 1:4:1. Nanoemulsion-based plant hexane extract formulations (NHEFs) were then produced by blending NHE-S : NHE-L at the following ratios: 4:0, 3:1, 2:2, 1:3 and 0:4, and labeled NHEF-1 through NHEF-5, respectively. All NHEs and NHEFs were diluted to a final concentration of 1.0% (w/v) in water and evaluated for mean particle size (hydrodynamic diameter), polydispersity index (PDI) and zeta potential using a Nano plus Zeta/Nano Particle Analyzer (Micromeritics Instrument Corporation, Japan) according to the manufacturer’s instructions.

In addition, the crude extracts demonstrating insecticidal activity were further characterized using a gas chromatography mass spectrometer/ mass spectrometer (GC-MS/MS) (Scion 436-GC MS/MS Triple Quad, Bruker, USA) equipped with an HP-5MS capillary column (30 m length x 0.25 mm I.D. x 0.25 μm film thickness) in splitless mode. The carrier gas (helium) flow rate was 1 mL min⁻¹, with a mass detection range of 30–500 amu. The oven temperature was programmed as follows: 50 °C for 2 min, increasing to 250 °C at 20 °C min⁻¹, then held at 250 °C for 23 min. The transfer line and ion source temperatures were maintained at 250 °C and 230 °C, respectively. Spectra were compared against the National Institute of Standards and Technology (NIST14) library, and compounds were identified when matching scores exceeded 85%.

### Insect culture

Sweet potato tubers (*Ipomoea batatas* (L.) *Lam* var. HRDI Sp-61) infested with a variety sweet potato weevil (*Cylas formicarius* Fabricius; SPW) were collected from Sa Kaeo Province, Thailand. Infested tubers were maintained in the laboratory at School of Agricultural Technology, KMITL, Thailand, using a method adapted from Ruddit et al.^11^. Adult (5–7 days old) from F_1_ to F_2_ laboratory-reared generations were used for all subsequent experimental testing.

### Insecticidal properties test

#### Non-nanoemulsion-based experiments

Preliminary screening in total, 21 botanical plant extracts (hexane, acetone, and ethanol fractions) were evaluated for toxicity against adult SPW at 5% (w/v). The initial screening focused on insecticidal activity, as SPW is considered the most critical pest in sweet potato fields, causing the greatest economic losses. A filter paper residue contact method, modified from Ruddit et al.^11^, was employed. Briefly, 1 mL of each extract solution was applied to a filter paper disc (Whatman™ No. 1) and placed in a 90 mm glass Petri dish. After allowing 3 min for solvent evaporation, 10 unsexed adult SPWs were released onto each disc, and the dish was sealed with Parafilm™. Experiments were conducted in a completely randomized design (CRD) with three replicates at 25 ± 2 °C. The experiment was conducted once independently, following preliminary runs to standardize the procedure under uniform laboratory conditions. Mortality was recorded at 24 h post-exposure and compared to a control (5% Tween-20 in water). The following mortality categories were used: very low (VL): <10%, low (L):10–25%, medium (M): 26–50%, high (H): 51–75% and very high (VH): >75%. Extracts rated as VH were selected for more detailed toxicity evaluation at 1−5% (w/v) using the same filter paper residue contact method. Mortality was recorded at 24 and 48 h after exposure. Lethal concentrations (LC_50_ and LC_90_) were calculated via probit analysis. Imidacloprid, applied at recommended field dose was used as the positive control.

#### Nanoemulsion-based experiments

NHEFs were assayed at 0.2-1.0% (w/v) against SPWs using the same filter paper residue protocol described above. Mortality was assessed at 24 and 48 h after exposure, and LC_50_ and LC_90_ values were determined. Co-toxicity coefficients (CTC) were calculated according to the equation described by Urvashi and Eswara Reddy^27^, and toxicity index (TI) were also determined according to Abbood et al.^28^.

The repellent activity, a modified filter paper residue test^11^, was used to assess repellency. Filter papers (Whatman™ No. 1) were halved; one half was treated with 0.1, 0.5, or 1.0% NHEF solutions, while the other half was treated with the corresponding control (Tween-20 in water at the same concentration). Each half received 0.5 mL of test solution, dried for 3 min, then reassembled in a 90 mm Petri dish. Ten unsexed adult SPWs were placed at the interface of the two halves, and the dish was sealed and held at 25 ± 2 °C. The number of insects on each half was recorded at 24 h. Experiments were performed in a CRD with three replicates. Repellency and attractancy percentages were classified as Doungnapa et al.^26^.

The oviposition inhibition, a modified tuber-dip assay^11^ was employed to evaluate oviposition. Sweet potato tubers (var. HRDI Sp-61) of uniform size were dipped in NHEF solutions at 0.1, 0.5, or 1.0% for 1 min, air-dried for 3 min, and placed into a 45 L ventilated plastic container. Three replicates (CRD) per concentration were tested. A total of 500 unsexed SPWs were released into each container and allowed 48 h for oviposition. Adult insects were subsequently removed, and the number of eggs deposited in/on the tubers was counted after 10 days. Oviposition inhibition rates were then calculated relative to the control (1% Tween-20 in water).

### Fungicidal properties test

A sweet potato tuber exhibiting dry rot symptoms was selected for fungal isolation, using a tissue transplantation technique adapted from Ferniah et al.^29^. Briefly, a 5 × 5 mm section was excised from the diseased area, surface-sterilized in 10% Clorox solution for 5 min, then rinsed in sterile distilled water for 10 min and air-dried on sterile tissue. The piece was placed onto potato dextrose agar (PDA) and incubated in a growth chamber (LAC-1075-N, Longyue, Shanghai, China) at 27 ± 2 °C, 80% RH, with a 12-hour light/dark cycle. Single colonies were sub-cultured onto fresh PDA to obtain pure isolates, which were characterized based on colony and cellular morphology. The isolate identified as *Fusarium* sp. was designated the master isolate for subsequent assays.

The poisoned food technique was employed to assess the impact of the NHEFs on fungal biomass production. NHEF solutions at 10, 20, and 30 ppm were prepared in 50 mL of potato dextrose broth (PDB) within 125 mL conical flasks; distilled water served as the control. A 6 mm agar plug taken from a 7-day-old culture of *Fusarium* sp. on PDA was transferred into each flask. Following 7 days of incubation at 27 ± 2 °C, the mycelial biomass was harvested through vacuum filtration (Whatman™ No. 1), oven-dried at 50 °C for 24 h, and weighed. All treatments were arranged in a CRD with three replicates. The percentage of fungal growth inhibition was calculated based on dry weight, following the method of Siripornvisal et al.^30^.

### Herbicidal properties test

Seeds of *A. tricolor* were purchased from Chia Tai Co., Ltd. and grown on a Thai farmer’s private land. *E. crus-galli* were collected from crop fields in Ladkrabang district, Bangkok, Thailand. After shade-drying for 3 months at room temperature, the seeds were further dried in an oven at 45 °C for 2 days to break dormancy. The pre-emergent herbicidal activity of the NHEFs was evaluated under laboratory conditions. The inhibition effect on seed germination and seedling growth of the tested seeds was evaluated using Petri dish assay. Glass Petri dishes (9 cm I.D.) were lined with double-sheet germination paper, were used for assay. Five milliliters of each NHEF treatment at 0.001, 0.01, 0.1, and 1.0% (w/v) were applied onto the germination paper. Twenty seeds of each test species were placed in separate Petri dishes, which were then sealed with Parafilm^®^ to minimize volatilization of the NHEFs. Distilled water served as a control. All treatments were arranged in a CRD, with five replicates per treatment. The dishes were incubated in a growth chamber (LAC-1075-N, Longyue, Shanghai, China) at 27 ± 2 °C, 80% RH, and a 12-hour light/dark cycle. After 7 days, survival rates as well as shoot and root lengths (cm) were measured. Herbicidal inhibition was calculated according to the method of Somala et al.^22^.

### Species identification and authentication

All organisms used in this study—including plant materials, insects, and associated fungi or weeds—were identified and authenticated by qualified experts in their respective fields. Plant species were verified by plant taxonomists; insect species (e.g., *Cylas formicarius*) were confirmed by entomologists based on standard morphological characteristics; whereas fungal and weed species were examined and validated by plant pathologists and weed scientists, respectively. This expert-based authentication approach ensured the taxonomic accuracy and reliability of all experimental materials used throughout the study. All plant, insect, fungal, and weed specimens examined in this study were properly preserved and deposited in the departmental reference collection of the School of Agricultural Technology, King Mongkut’s Institute of Technology Ladkrabang (KMITL), Bangkok, Thailand. Voucher specimen codes are archived in the collection and are available upon request.

## Results

### Preliminary screening of plant extracts

At an application rate of 5% (w/v), the hexane and acetone extract of star anise and long pepper, as well as the hexane extract of kratom, demonstrated very high contact toxicity to adult SPWs (> 75% mortality) within 24 h. The positive control, imidacloprid at the recommended field dose, induced high mortality, thereby validating the reliability of the bioassay system (Table 1). In addition, the hexane extracts of myrtle grass, Siamese’s Maerua, clove, the acetone extract of clove, and the ethanol extracts of star anise and long pepper produced high mortality rates ranging from 51 to 75%. Notably, the hexane extracts of star anise and long pepper exhibited the greatest toxicity, with LC_50_ values of 2.051 and 2.415% at 24 h post-treatment and 0.881 and 1.662% at 48 h post-treatment, respectively. Subsequent ranking placed the hexane extract of kratom and the acetone extracts of star anise and long pepper in the next tier of efficacy (LC_50_ = 3.852–5.071% at 24 h; 2.159–3.269% at 48 h) (Table 2).

### Chemical composition of potent extracts

Given their superior toxicity profiles, the hexane extracts of star anise and long pepper were subjected to GC-MS/MS analysis. The major constituent of the star anise extract was anethole (84.49%), while that of the long pepper extract was composed of cis-2-methyl-7-octadecene (12.14%), germacrene D (10.72%), eicosane (9.30%), α-humulene (9.29%), docosane (8.72%), caryophyllene (7.74%), β-bisabolene (6.03%), and 9-heptadecanol (5.11%) (Table 3).

### Nanoemulsion characterization (NHEs and NHEFs)

The mean droplet size of NHE-S (star anise), NHE-L (long pepper), and their combinations (NHEF-1 to NHEF-5) in water (1.0%, w/v) ranged from 17.90 to 22.10 nm (Table 4). Polydispersity index (PDI) values spanned 0.16–0.25, suggesting a narrow size distribution. The zeta potentials of these systems were negative (− 8.85 to − 1.27 mV), indicating moderate colloidal stability.

### Insecticidal efficacy of nanoemulsion-based formulations

All NHEFs demonstrated markedly greater contact toxicity against SPWs than their non-nanoemulsified counterparts. The toxicity index (TI) of NHEF-1 and NHEF-5 was 18.0- and 16.1-fold higher, respectively, compared to the corresponding crude extracts at 24 h post-application. NHEF-1 and NHEF-5 had LC_50_​ values of 0.114 and 3.02%, respectively, at 24 h, decreasing to 0.093 and 0.138% at 48 h. Varying the ratio of star anise (NHE-S) to long pepper (NHE-L) extracts within each NHEF formulation revealed that NHEF-2 (3:1 ratio) was the most potent (LC_50_ = 0.094% at 24 h), with a co-toxicity coefficient (CTC) of 121.3. By contrast, formulations containing higher proportions of NHE-L displayed reduced toxicity (CTC = 76.0–91.9) (Table 5).

### Repellency assays

**Fig. 1:**
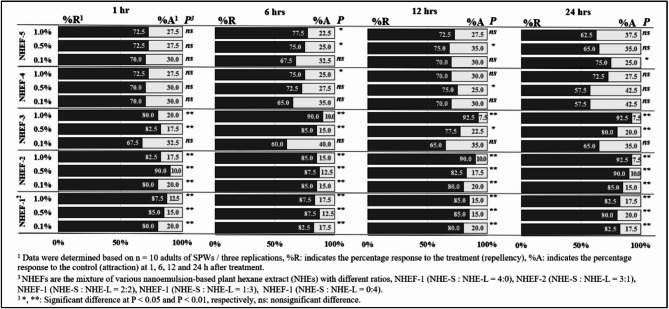
Response percentage (repellency and attraction) of sweet potato weevil (*Cylas formicarius*; SPW) to different concentrations of nanoemulsion-based plant hexane extract formulations (NHEFs) via contact method at 1, 6, 12 and 24 h after treatment.

At concentrations of 0.1, 0.5, and 1.0%, both NHEF-1 and NHEF-2 provided > 80% repellency against adult SPWs, significantly exceeding (*P* < 0.01) the repellent effect of the control. These two formulations consistently outperformed the remaining NHEFs over the full concentration range tested (Fig. 1).

### Oviposition Inhibition

**Fig. 2:**
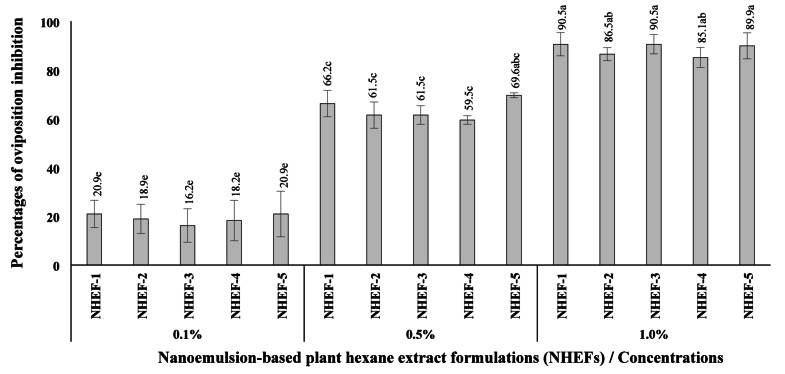
Oviposition inhibition percentage of different concentrations of nanoemulsion-based plant hexane extract formulations (NHEFs) against sweet potato weevil (*Cylas formicarius*; SPW), determined via tuber-dipping method. Each NHEFs is a mixture of star anise (NHE-S) and long pepper (NHE-L) nanoemulsion-based hexane extracts at various ratios: NHEF-1 (NHE-S : NHE-L = 4:0), NHEF-2 (NHE-S : NHE-L = 3:1), NHEF-1 (NHE-S : NHE-L = 2:2), NHEF-1 (NHE-S : NHE-L = 1:3), NHEF-1 (NHE-S : NHE-L = 0:4); Different letters indicate significant differences among treatments (*P* < 0.05), as determined by DMRT.

The capacity of NHEFs to inhibit oviposition on sweet potato tubers was evaluated at 0.1, 0.5, and 1.0%. All formulations at 1.0% inhibited egg deposition by 85.1–90.5%, while at 0.5%, they achieved 59.5–69.6% inhibition. The lowest concentration (0.1%) resulted in comparatively minimal inhibition (< 20.9%) (Figs. 2 and Figure S1).

### Fungicidal activity

**Fig. 3:**
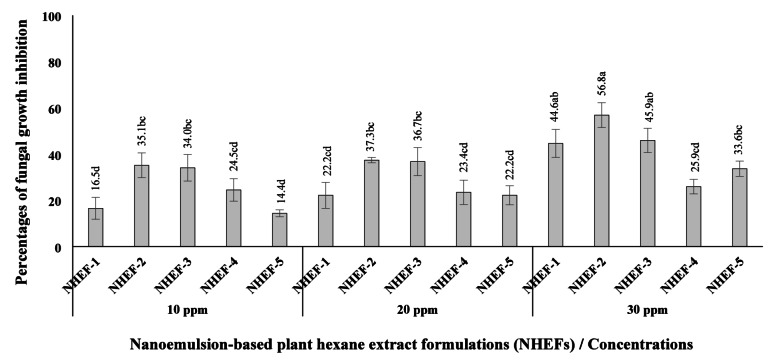
Fungal inhibition percentage of different concentrations of nanoemulsion-based plant hexane extract formulations (NHEFs) against *Fusarium* sp. in potato dextrose broth (PDB) medium. Each NHEFs is a mixture of star anise (NHE-S) and long pepper (NHE-L) nanoemulsion-based extracts at various ratios: NHEF-1 (NHE-S : NHE-L = 4:0), NHEF-2 (NHE-S : NHE-L = 3:1), NHEF-1 (NHE-S : NHE-L = 2:2), NHEF-1 (NHE-S : NHE-L = 1:3), NHEF-1 (NHE-S : NHE-L = 0:4); Different letters indicate significant differences among treatments (*P* < 0.05), as determined by DMRT.

Against *Fusarium* sp. in PDB, NHEF-2 at 30 ppm exhibited the highest inhibition of fungal growth (56.8%), followed by NHEF-1 and NHEF-3 (44.6–45.9%). Other formulations provided < 34% inhibition at 30 ppm. Across lower concentrations (10–20 ppm), fungal suppression in all treatments did not exceed 38% (Fig. 3 and Figure S2).

### Herbicidal activity

All NHEFs exerted stronger inhibitory effects on *A. tricolor* than on *E. crus-galli*, completely obstructing seed germination at 0.01 and 1.0%, respectively (Table 6). At 0.001%, *A. tricolor* survival remained 60.0–67.5% of control values, with corresponding reductions in shoot and root length (71.0–78.4% and 80.1–82.1% of control, respectively). In contrast, *E. crus-galli* survival at 0.1% was 74.6–89.3% of control, accompanied by shoot and root lengths of 16.4–50.1% and 37.0–49.5% of control, respectively.

## Discussion

The present study demonstrated that hexanolic extracts of star anise and long pepper exerted potent insecticidal activity against SPW, specifically in terms of mortality, repellency, and oviposition inhibition. Compared to acetone and ethanol extracts, the hexane extracts consistently exhibited higher efficacy. These findings are consistent with several previous studies showing that non-polar solvents (e.g., hexane) often extract bioactive compounds more effectively, thus resulting in stronger insecticidal potential^31,32^. The inclusion of imidacloprid as a positive control, which consistently produced high mortality, confirmed the reliability of the bioassay system.

A major contributing factor to the efficacy of star anise’s hexanolic extract is its high *trans*-anethole content (84.49%), which was identified as the principal bioactive compound responsible for insecticidal properties against SPW. This enhanced activity may be attributed to trans-anethole’s ability to disrupt insect neural transmission and membrane integrity, as reported in previous studies^33−35^. This observation aligns with Choi et al.^32^, who reported that *trans*-anethole constituted 94.24% of the hexane fraction of star anise, demonstrating repellency against *Plodia interpunctella* larvae. Wang et al.^31^ further confirmed that *trans*-anethole exhibited effective fumigant activity, achieving 100% mortality of the rusty grain beetle (*Cryptolestes ferrugineus*) at 30 mL/L for 24 h. Additionally, *trans*-anethole and related anethole derivatives have demonstrated broad-spectrum activity against various insects and mites^36,37^. Pumnuan et al.^38^ similarly showed that star anise hexanolic extract containing 99.5% *trans*-anethole had higher toxicity against bruchid beetles (*Callosobruchus maculatus* and *C. chinensis*) than acetone and ethanol extracts, while also preserving mung bean seed germination at 1% coating over a 6-month storage period.

In contrast, while the hexanolic extract of long pepper also showed strong insecticidal effects, no single compound appeared in a notably high concentration. Instead, over 70 bioactive components—alkaloids, terpenoids, lignans, flavones, propenylphenols, kawapyrones, and dihydrochalcones—have been identified in its purified fractions^38^. These collective constituents may act synergistically, contributing to the extract’s toxicity. Earlier research corroborates this notion, with the hexane extract of long pepper showing potent activity against *Spodoptera litura* larvae (LD_50_ = 0.87 µg/larva at 24 h)^39^. Interestingly, the crude hexanolic extract demonstrated stronger activity compared to isolated compounds, suggesting that multiple constituents in combination are likely required to achieve full insecticidal potential. Such findings suggest synergistic interactions among the diverse phytochemicals in long pepper, collectively enhancing its overall toxicity beyond that of individual compounds. Beyond the activity of the pure extracts, the current research also highlighted the enhanced efficacy of NHEFs against SPW. These nanoemulsions were 16–18-fold more effective than their non-nanoemulsified counterparts. Previous studies have reported that nano-formulations improve the solubility, stability, and bioavailability of active ingredients, thereby increasing insecticidal efficacy^40,41^. The significantly larger surface area of the nanoemulsions promotes better release and absorption of the active compounds, and the incorporation of surfactants further enhances kinetic stability^42^. Furthermore, the nanoemulsion system increased bioavailability and persistence of the active constituents, thereby contributing to the increased efficacy observed against SPW, consistent with previous reports^42−45^. Nanoemulsions have likewise been documented to possess potent antifungal and herbicidal properties, often outperforming non-nano formulations due to their superior penetration through pest cuticles or plant tissues^26,43−47^. The most active extracts, particularly star anise and long pepper, were prioritized for nanoemulsion formulation and subsequently evaluated for fungicidal and herbicidal activities. In addition to insect control, the nanoemulsion-based formulations in this study demonstrated substantial antifungal activity against *Fusarium* sp. at low concentrations (30 ppm), inhibiting fungal growth by more than 56%. Similar results have been reported with nanoemulsion-based essential oils from thyme, sweet basil, clove, and lemongrass, which inhibited *Fusarium oxysporum* and reduced wilt disease without harming seed germination^48,49^. These antifungal effects may stem from increased membrane permeability and leakage of intracellular constituents caused by compounds such as anethole and related phenylpropanoids^33−35^.

Finally, the results revealed that nanoemulsion-based herbicidal formulations effectively inhibited weed growth parameters, with *A. tricolor* showing a higher susceptibility than *E. crus-galli*. Complete germination inhibition was achieved at 0.01 and 1.0% for *A. tricolor* and *E. crus-galli*, respectively. Previous work supports that nanoemulsion systems enhance herbicide penetration and activity^42,50^. Citronella-based nanoemulsions, for instance, have been shown to inhibit weed seed germination as a natural pre-emergence herbicide due to their tiny particle size. Therefore, after being applied to weed seeds or leaf surfaces, nanoemulsions have been developed to enhance the characteristics of essential active chemicals, ensuring their efficient release as well as rapid interaction with plant cells^22^.

Taken together, these findings suggest that hexanolic extracts from star anise and long pepper—especially in nanoemulsion-based formulations—offer promising, broad-spectrum pest management solutions. Their efficacy encompasses insecticidal, antifungal, and herbicidal activities, highlighting the potential for eco-friendly, multi-functional crop protection strategies. Further work to optimize formulations, investigate synergistic interactions among minor constituents, and evaluate large-scale field performance will help maximize the benefits of these plant-based nanoemulsions in sustainable agriculture farming.

## Conclusions

This study concludes that plant extract-based nanoemulsion formulas (NHEFs) containing star anise and long pepper hexanolic extracts significantly enhance pest control in sweet potato, providing an eco-friendly alternative to conventional pesticides. At 0.5−1.0% of NHEFs or 0.1−0.2% for the hexanolic extracts, these treatments were highly effective against *Cylas formicarius*, *Fusarium* sp., *Amaranthus tricolor* and *Echinochloa crus-galli*. As bio-pesticides, they are safer for users and more environmentally friendly, representing a notable advancement in sustainable agriculture.

These findings suggest that nanoemulsion formulations can be practically integrated into pest and weed management programs for sweet potato cultivation. Their eco-friendly profile also highlights their potential as sustainable alternatives to conventional pesticides, reducing chemical reliance while maintaining effective control under field conditions.

**Table 1:** Contact toxicity of hexane, acetone, and ethanol extracts from two botanical species against sweet potato weevil (*Cylas formicarius*; SPW) at 24 h after treatment.

Family / Category	Common name	Scientific name	Part used	Solvent Extraction	Effecacy^1^ (24 h after treatment)
Acoraceae	Myrtle grass	*Acorus calamus*	Roots and rhizomes	Hexane	L
				Acetone	VL
				Ethanol	VL
Asteraceae	Bitter bush	*Chromolaena odorata*	Leaves	Hexane	VL
				Acetone	VL
				Ethanol	VL
Schisandraceae	Star anise	*Illicium verum*	Flower	Hexane	VH
				Acetone	VH
				Ethanol	L
Capparaceae	Siamese’s Maerua	*Maerua siamensis*	Leaves	Hexane	L
				Acetone	VL
				Ethanol	VL
Fabaceae	Snake climber	*Bauhinia scandens*	Vine	Hexane	VL
				Acetone	VL
				Ethanol	VL
	Golden shower	*Cassia fistula*	Pod	Hexane	VL
				Acetone	VL
				Ethanol	VL
	Sasswood	*Erythrophleum succirubrum*	Leaves	Hexane	VL
				Acetone	VL
				Ethanol	VL
	Tuba root	*Derris elliptica*	Roots and rhizomes	Hexane	VL
				Acetone	VL
				Ethanol	VL
	Jewel vine	*Derris scandens*	Vine	Hexane	VL
				Acetone	VL
				Ethanol	VL
Rubiaceae	Kratom	*Mitragyna speciosa*	Leaves	Hexane	VH
				Acetone	VL
				Ethanol	VL
Lamiaceae	Mint weed	*Hyptis suaveolens*	Leaf, flower and stem	Hexane	VL
				Acetone	VL
				Ethanol	VL
	Cinnamon	*Cinnamomum bejolghota*	Bark	Hexane	VL
				Acetone	VL
				Ethanol	VL
Verbebaceae	Weeping lantana	*Lantana camara*	Leaf, flower and stem	Hexane	VL
				Acetone	VL
				Ethanol	VL
Stemmonaceae	Stemona	*Stemona tuberosa*	Rhizome	Hexane	VL
				Acetone	VL
				Ethanol	VL
Myrtaceae	Clove	*Syzygium aromaticum*	Buds	Hexane	L
				Acetone	L
				Ethanol	VL
	Blue gum	*Eucalyptus camaldulensis*	Leaves	Hexane	VL
				Acetone	VL
				Ethanol	VL
Piperaceae	Long pepper	*Piper retrofractum*	Fruit	Hexane	VH
				Acetone	VH
				Ethanol	L
Menispermaceae	Heart-leaved moonseed	*Tinospora crispa*	Stem	Hexane	VL
				Acetone	VL
				Ethanol	VL
Meliaceae	Siamese neem tree	*Azadirachta indica*	Leaves	Hexane	VL
				Acetone	VL
				Ethanol	VL
Zingiberaceae	Siam cardamom	*Amomum krervanh*	Roots	Hexane	VL
				Acetone	VL
				Ethanol	VL
	Cassumunar ginger	*Zingiber montanum*	Rhizome	Hexane	VL
				Acetone	VL
				Ethanol	VL
Synthetic insecticide	Imidacloprid	−	−	−	VH

^1^Classification of mortality; VL: Very low < 10%, L: Low 10–25%, M: Medium 26–50%, High 51–75% and VH: Very high > 75%.

**Table 2:** The LC_50_ and LC_90_ of plant hexane extracts (PHEs) against sweet potato weevil (*Cylas formicarius*; SPW) at 24 and 48 h after treatment (contact method).

Treatment^2^	After treated (h)	Toxicity^1^			SE	χ^2^	*P* ^4^
		Regression^3^	LC_50_ (% of PHE)	LC_90_ (% of PHE)			
SH	24	Y = 0.598x – 1.227	2.051	4.193	0.043	31.213	< 0.001^**^
	48	Y = 1.640x – 1.445	0.881	1.663	0.145	36.624	< 0.001^**^
SA	24	Y = 0.377x – 1.452	3.852	7.251	0.037	16.190	0.003^**^
	48	Y = 0.634x – 1.368	2.159	4.182	0.045	30.445	< 0.001^**^
LH	24	Y = 0.505x – 1.219	2.415	4.954	0.038	22.366	< 0.001^**^
	48	Y = 0.473x – 0.787	1.662	4.368	0.038	61.494	< 0.001^**^
LA	24	Y = 0.378x – 1.914	5.071	8.466	0.042	5.658	0.226^ns^
	48	Y = 0.422x – 1.381	3.269	6.303	0.037	17.949	0.001^**^
KH	24	Y = 0.581x – 2.034	3.499	5.703	0.044	12.385	0.015^*^
	48	Y = 0.568x – 1.636	2.879	5.135	0.041	8.69	0.690^ns^

^1^Data were determined based on n = 10 adults of SPWs / three replications lethal concentrations of plant hexane extract (PHE) needed to kill 50% and 90% of the insects (LC_50_ and LC_90_, respectively) at 24 and 48 h after treatment. ^2^Hexane extracts from star anise, long pepper and kratom (SH, LH and KH, respectively), and acetone extracts from star anise and long pepper (SA, and LA, respectively). ^3^Probit (Y) = Intercept + Slope × (concentration: x). ^4^*, **: Significant difference at *P* < 0.05 and *P* < 0.01, respectively, ns: nonsignificant difference.

**Table 3:** Main components in hexanolic extracts of star Anise and long pepper analyzed by GC-MS/MS.

Star anise		Long pepper	
Chemical components	%	Chemical components	%
*trans*-Anethole	84.49	*cis*-2-Methyl-7-octadecene	12.14
Foeniculin	3.28	Germacrene D	10.72
Limonene	3.18	Eicosane	9.30
Estragole	2.01	α-Humulen	9.29
Bergamotene	1.65	Docosane	8.72
Anisaldehyde	1.63	Caryophyllene	7.74
other compounds	3.76	β-Bisabolene	6.03
		9-Heptadecanol (CAS)	5.11
		(2E,4E,10E)-N-Isobutylhexadeca-2,4,10-trienamide	4.07
		Sabinene	3.77
		α-bisabolene	3.42
		(2E,4E,14E)-N-Isobutylicosa-2,4,14-trienamide	2.56
		Piperine	2.52
		2-Methyl-Z, Z-3,13-octadecadienol	2.35
		Tetradecane-Dup1	1.74
		*trans*-β-Farnesene	1.13
		Piperanine	1.06
		other compounds	8.33

**Table 4:** Particle size, polydispersity index (PDI) and zeta potential of nanoemulsion-based plant hexane extract (NHEs) and nanoemulsion-based plant hexane extract formulations (NHEFs) at 1.0% in water.

Nanoemulsions (1.0% in water)	Particle size (nm)	PDI	Zeta Potential (mV)
***Nanoemulsion-based plant hexane extract (NHEs)***, *(% of plant hexane extract: PHE)*			
NHE-S (Star anis hexane extract : NP9 : Tween 20 = 1:3:1), *(0.20% of PHE)*	17.90 ± 2.94	0.17 ± 0.16	-1.33 ± 0.94
NHE-L (Long pepper hexane extract : NP9 : Tween 20 = 1:4:1), *(0.16% of PHE)*	24.64 ± 2.23	0.18 ± 0.06	-6.65 ± 1.06
***Nanoemulsion-based plant hexane extract formulations (NHEFs)***			
NHEF-1 (NHE-S : NHE-L = 4:0), *(0.20% of PHE)*	17.90 ± 2.94	0.17 ± 0.16	-1.33 ± 0.94
NHEF-2 (NHE-S : NHE-L = 3:1), *(0.19% of PHE)*	22.10 ± 1.12	0.25 ± 0.07	-1.27 ± 0.66
NHEF-3 (NHE-S : NHE-L = 2:2), *(0.18% of PHE)*	21.60 ± 1.79	0.20 ± 0.23	-1.80 ± 1.06
NHEF-4 (NHE-S : NHE-L = 1:3), *(0.17% of PHE)*	20.88 ± 0.28	0.19 ± 0.03	-8.85 ± 0.97
NHEF-5 (NHE-S : NHE-L = 0:4), *(0.16% of PHE)*	24.64 ± 2.23	0.16 ± 0.03	-6.65 ± 1.06

**Table 5:** Contact toxicity (LC_50_ and LC_90_), toxicity index (TI) and co-toxicity coefficient (CTC) of non-nanoemulsion-based (NNE) and nanoemulsion-based (NE) plant hexane extract formulations (PHEFs) against sweet potato weevil (*Cylas formicarius*; SPW) at 24 and 48 h after treatment.

NHEFs / after treatment (h)	Non-nanoemulsion-based		Nanoemulsion-based				Toxicity Index (TI)	Co-Toxicity Coefficient (CTC)
	Toxicity^1^ (% of PHE)		Toxicity^1^ (% of NHEF)		Toxicity^1^ (% of PHE)			
	LC_50_ (%)	LC_90_ (%)	LC_50_ (%)	LC_90_ (%)	LC_50_ (%)	LC_50_ (%)		
NHEF-1 (NHE-S)								
24	2.051	4.193	0.570	1.065	0.114	0.213	18.0	100.0
48	0.881	1.663	0.464	0.889	0.093	0.178	9.5	100.0
NHEF-2 (NHE-S: NHE-L = 3:1)								
24	-	-	0.470	0.924	0.094	0.185	-	121.3
48	-	-	0.377	0.826	0.075	0.165	-	124.0
NHEF-3 (NHE-S: NHE-L = 2:2)								
24	-	-	0.622	1.333	0.124	0.267	-	91.9
48	-	-	0.538	1.204	0.108	0.241	-	86.1
NHEF-4 (NHE-S: NHE-L = 1:3)								
24	-	-	0.693	1.288	0.139	0.258	-	82.0
48	-	-	0.606	1.181	0.121	0.236	-	76.9
NHEF-5 (NHE-L)								
24	2.415	4.954	0.750	1.511	0.150	0.302	16.1	76.0
48	1.662	4.368	0.691	1.486	0.138	0.297	12.0	67.4

^1^Data were determined based on n = 10 adults of SPWs / three replications lethal concentrations of non-nanoemulsion-based and nanoemulsion-based plant hexane extract formulations needed to kill 50% and 90% of the insects (LC_50_ and LC_90_, respectively) at 24 and 48 h after treatment. ^2^NHEFs are the mixture of various nanoemulsion-based plant hexane extract (NHEs) with different ratios, NHEF-1 (NHE-S : NHE-L = 4:0), NHEF-2 (NHE-S : NHE-L = 3:1), NHEF-1 (NHE-S : NHE-L = 2:2), NHEF-1 (NHE-S : NHE-L = 1:3), NHEF-1 (NHE-S : NHE-L = 0:4).

**Table 6:** Inhibitory effect of nanoemulsion-based plant hexane extract formulations (NHEFs) on survival rate and shoot and root length of *Amaranthus tricolor* and *Echinochloa crus-galli* at 7 days after treatments in a Petri-dish test.

NHEFs^1^	Concentrations (%)	Amaranthus tricolortricolor			Echinochloa crus-galli		
		Survival rate (% of control)	Shoot length (% of control)	Root length (% of control)	Survival rate (% of control)	Shoot length (% of control)	Root length (% of control)
NHEF-1	0.001	67.5 ± 13.9	78.4 ± 14.2	80.9 ± 12.2	-	-	-
	0.01	0.0 ± 0.0	-	-	92.5 ± 8.9	72.0 ± 5.6	77.8 ± 2.6
	0.1	-	-	-	88.0 ± 6.4	50.1 ± 12.5	37.0 ± 1.8
	1	-	-	-	0.0 ± 0.0	-	-
NHEF-2	0.001	65.0 ± 12.0	76.9 ± 13.8	81.7 ± 4.3	-	-	-
	0.01	0.0 ± 0.0	-	-	97.5 ± 4.6	71.1 ± 9.8	73.8 ± 2.6
	0.1	-	-	-	84 ± 10.9	37.0 ± 10.9	41.7 ± 5.0
	1	-	-	-	0.0 ± 0.0	-	-
NHEF-3	0.001	67.5 ± 11.6	72.7 ± 7.9	82.1 ± 10.5	-	-	-
	0.01	0.0 ± 0.0	-	-	97.5 ± 4.6	71.8 ± 3.5	75.2 ± 4.4
	0.1	-	-	-	89.3 ± 6.2	31.9 ± 6.8	45.1 ± 4.5
	1	-	-	-	0.0 ± 0.0	-	-
NHEF-4	0.001	62.5 ± 13.9	71.0 ± 4.0	81.7 ± 10.3	-	-	-
	0.01	0.0 ± 0.0	-	-	97.5 ± 4.6	74.1 ± 2.8	72.5 ± 2.3
	0.1	-	-	-	74.6 ± 10.7	18.8 ± 3.3	46.9 ± 3.3
	1	-	-	-	0.0 ± 0.0	-	-
NHEF-5	0.001	60.0 ± 7.6	73.0 ± 8.6	80.1 ± 13.3	-	-	-
	0.01	0.0 ± 0.0	-	-	95.0 ± 5.3	73.2 ± 2.9	71.3 ± 3.2
	0.1	-	-	-	77.3 ± 10.3	16.4 ± 2.8	49.5 ± 3.2
	1	-	-	-	0.0 ± 0.0	-	-

^1^NHEFs are the mixture of various nanoemulsion-based plant hexane extract (NHEs) with different ratios, NHEF-1 (NHE-S : NHE-L = 4:0), NHEF-2 (NHE-S : NHE-L = 3:1), NHEF-1 (NHE-S : NHE-L = 2:2), NHEF-1 (NHE-S : NHE-L = 1:3), NHEF-1 (NHE-S : NHE-L = 0:4).

## Supplementary Information

Below is the link to the electronic supplementary material.

Supplementary Material 1

## Data Availability

All data generated or analyzed during this study are included in this published article.
