# Supplementary material for: Plant extract-based nanoemulsion for controlling sweet potato pests and weeds
Source: Sci Rep. 2025 Dec 10;16:2565. doi: 10.1038/s41598-025-32263-9 (PMC12820081; doi:10.1038/s41598-025-32263-9)
Supplement: Supplementary file 1 — Supplementary Material 1 [file 41598_2025_32263_MOESM1_ESM.docx]

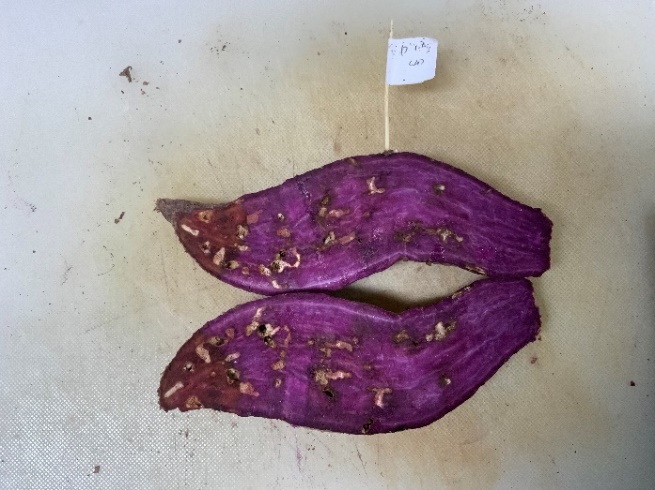

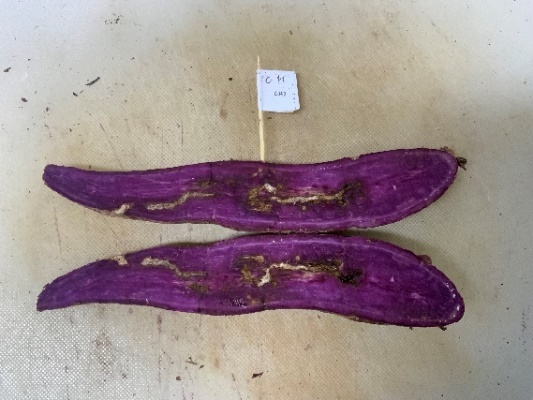

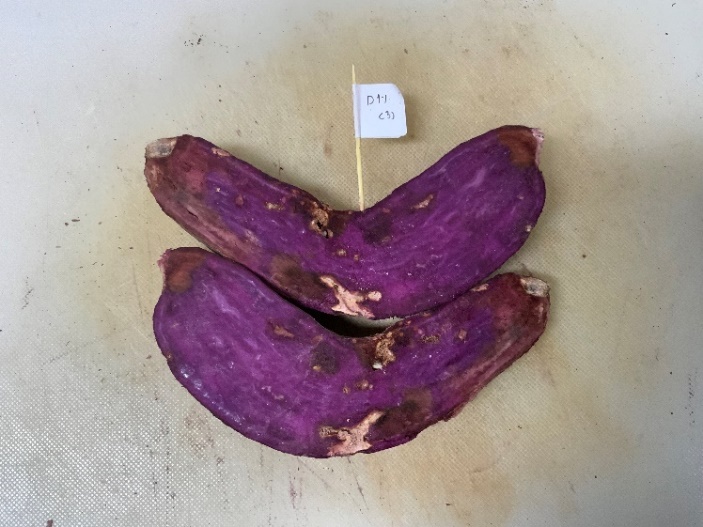

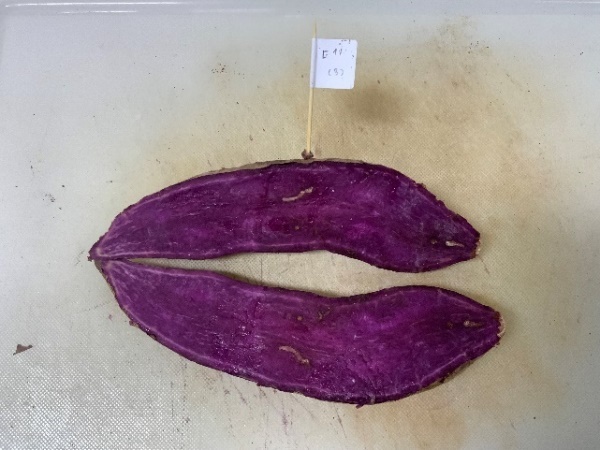


D

C

B

A

**Figure S1.** Damage characteristics of sweet potatoes infested by the sweet potato weevil (*Cylas formicarius*) after treatment with NHEF-2 at different concentrations, using the tuber-dipping method. (A) Control, (B) NHEF-2 (0.1%), (C) NHEF-2 (0.5%), (D) NHEF-2 (1.0%).

NHEF-2 is a nanoemulsion-based plant hexane extract formulation compose of star anise (NHE-S) and long pepper (NHE-L) at a 3:1 ratio.


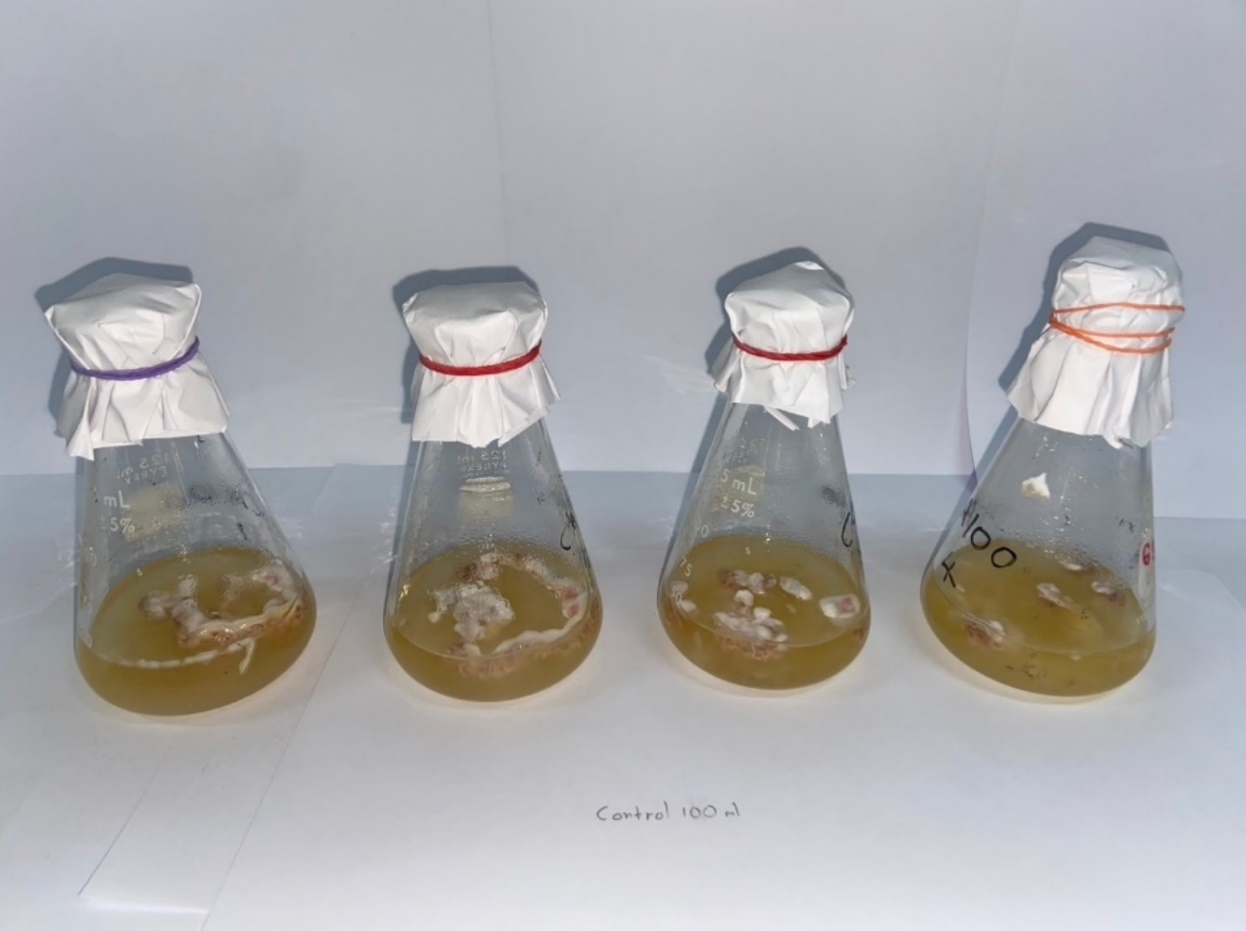

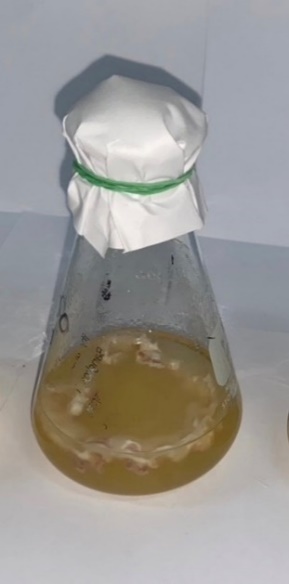

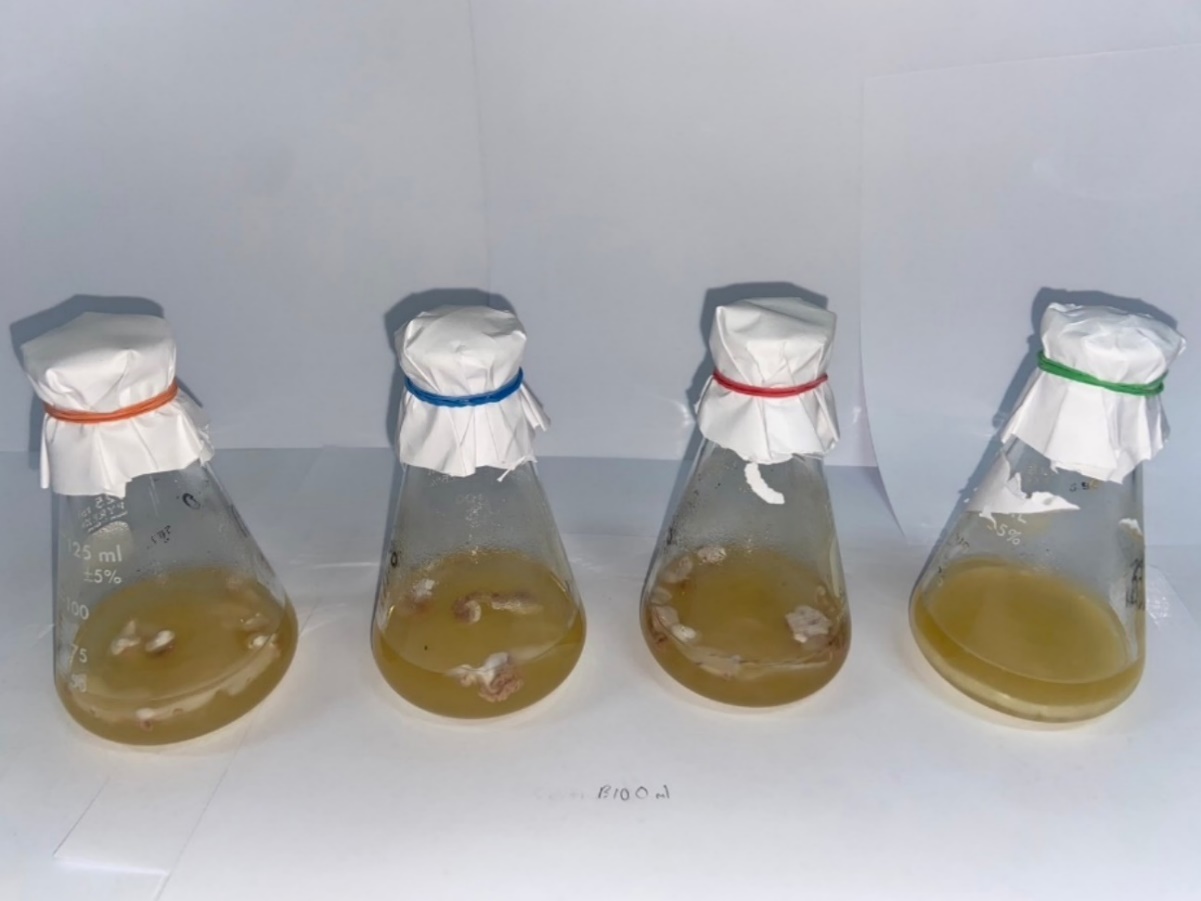

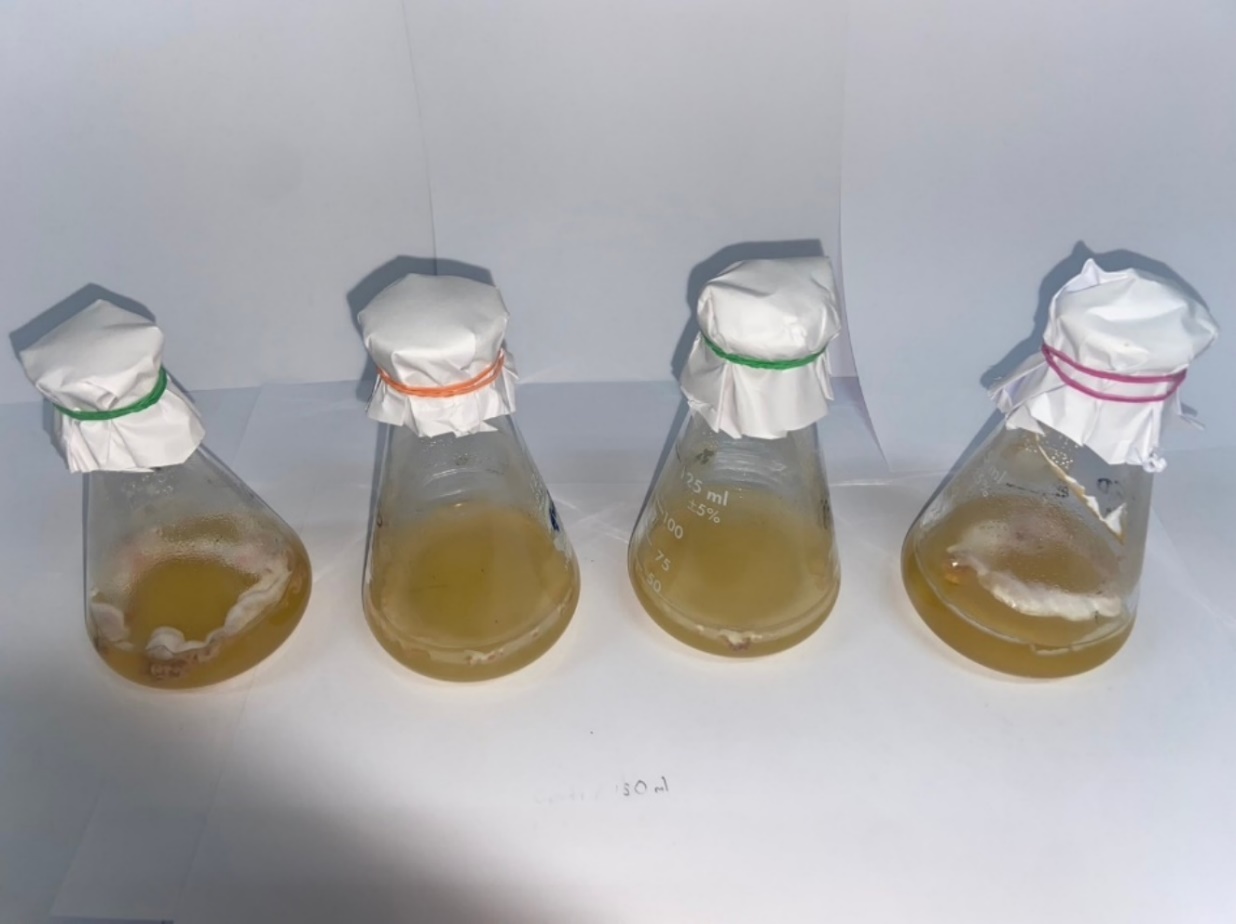


D

C

B

A

**Figure S2.** Morphological characteristics of *Fusarium* sp. In potato dextrose broth (PDB) medium under a poisoned food technique after treatment with NHEF-2 at different concentrations: (A) Control, (B) NHEF-2 (10 ppm), C: NHEF-2 (20 ppm), D: NHEF-2 (30 ppm).

NHEF-2 is a nanoemulsion-based plant hexane extract formulation consisting of star anise (NHE-S) and long pepper (NHE-L) in a 3:1 ratio.
